# Hybrid Dual-Context Prompted Cross-Attention Framework with Language Model Guidance for Multi-Label Prediction of Human Off-Target Ligand–Protein Interactions

**DOI:** 10.3390/ijms27021126

**Published:** 2026-01-22

**Authors:** Abdullah, Zulaikha Fatima, Muhammad Ateeb Ather, Liliana Chanona-Hernandez, José Luis Oropeza Rodríguez

**Affiliations:** 1 Center for Computing Research, Instituto Politécnico Nacional, Mexico City 07320, Mexico; abdullah2025@cic.ipn.mx (A.); 03-134211-022@student.bahria.edu.pk (M.A.A.); 2 Department of Computer Sciences, Bahria University, Lahore 54600, Pakistan; 3 Faculty of Allied Health Sciences, Superior University, Lahore 54000, Pakistan; su91-bmitm-f23-248@superior.edu.pk; 4 Zacatenco Unit, Higher School of Mechanical and Electrical Engineering, Instituto Politécnico Nacional, Mexico City 07700, Mexico; lchanona@gmail.com

**Keywords:** cross-attention, deep learning, drug discovery, graph transformer, model interpretability, multimodal representation, off-target prediction, polypharmacology, protein–ligand interactions, scaffold generalisation

## Abstract

Accurately identifying drug off-targets is essential for reducing toxicity and improving the success rate of pharmaceutical discovery pipelines. However, current deep learning approaches often struggle to fuse chemical structure, protein biology, and multi-target context. Here, we introduce HDPC-LGT (Hybrid Dual-Prompt Cross-Attention Ligand–Protein Graph Transformer), a framework designed to predict ligand binding across sixteen human translation-related proteins clinically associated with antibiotic toxicity. HDPC-LGT combines graph-based chemical reasoning with protein language model embeddings and structural priors to capture biologically meaningful ligand–protein interactions. The model was trained on 216,482 experimentally validated ligand–protein pairs from the Chemical Database of Bioactive Molecules (ChEMBL) and the Protein–Ligand Binding Database (BindingDB) and evaluated using scaffold-level, protein-level, and combined holdout strategies. HDPC-LGT achieves a macro receiver operating characteristic–area under the curve (macro ROC–AUC) of 0.996 and a micro F1-score (micro F1) of 0.989, outperforming Deep Drug–Target Affinity Model (DeepDTA), Graph-based Drug–Target Affinity Model (GraphDTA), Molecule–Protein Interaction Transformer (MolTrans), Cross-Attention Transformer for Drug–Target Interaction (CAT–DTI), and Heterogeneous Graph Transformer for Drug–Target Affinity (HGT–DTA) by 3–7%. External validation using the Papyrus universal bioactivity resource (Papyrus), the Protein Data Bank binding subset (PDBbind), and the benchmark Yamanishi dataset confirms strong generalisation to unseen chemotypes and proteins. HDPC-LGT also provides biologically interpretable outputs: cross-attention maps, Integrated Gradients (IG), and Gradient-weighted Class Activation Mapping (Grad-CAM) highlight catalytic residues in aminoacyl-tRNA synthetases (aaRSs), ribosomal tunnel regions, and pharmacophoric interaction patterns, aligning with known biochemical mechanisms. By integrating multimodal biochemical information with deep learning, HDPC-LGT offers a practical tool for off-target toxicity prediction, structure-based lead optimisation, and polypharmacology research, with potential applications in antibiotic development, safety profiling, and rational compound redesign.

## 1. Introduction

Accurate prediction of protein–ligand interactions is a foundational aspect of computational drug discovery, supporting rational drug design, off-target risk assessment, and toxicity profiling [1,2]. As modern therapeutics increasingly require multi-target understanding, computational frameworks must capture biochemical fidelity while maintaining strong generalisation across heterogeneous ligands and diverse protein families [3,4].

Traditional computational approaches, including molecular coupling and quantitative structure–activity relationship (QSAR) modelling, have contributed to drug design but rely on hand-crafted descriptors and show limited flexibility and generalisation [5]. Deep learning models such as DeepDTA learn richer molecular representations, yet they often lack sufficient contextual reasoning to resolve complex binding dependencies [6]. Graph-based frameworks, including GraphDTA and Self-Attention Graph with Cross-Target Attention (SAG-CTA), further improve chemical structure modelling but typically encode proteins in simplified forms, limiting cross-reactivity prediction [7,8].

Transformer-based methods such as MolTrans, TransformerCPI, and CAT-DTI introduce attention-driven sequence and structural reasoning; however, most remain constrained to single-label predictions and show reduced generalisation to unseen ligands and proteins [9,10,11]. More recently, advances in protein language models such as Evolutionary Scale Modeling 2 (ESM-2) and ProtBert and developments in graph neural networks have opened opportunities for unified modelling of sequence, structure, and spatial biochemical context [12,13]. Nevertheless, current systems often struggle to integrate heterogeneous molecular modalities or incorporate biochemical priors such as contact maps and docking energies, which limits interpretability and restricts biophysically grounded off-target prediction [14,15].

To address these challenges, we introduce HDPC-LGT, a hybrid dual-prompt cross-attention transformer designed for multi-label ligand–protein interaction prediction across sixteen human targets. The framework aims to model realistic polypharmacology by capturing simultaneous off-target behaviour rather than isolated ligand–protein pairs. HDPC-LGT leverages hierarchical ligand graphs, protein sequence/structure representations, and interaction-specific cross-attention to enable robust and interpretable drug–target modelling. Importantly, this architecture is positioned for application within broader drug discovery pipelines, supporting off-target toxicity screening, prioritisation of candidate scaffolds, and structure-based therapeutic refinement.

Key Contributions of This Work:

(a) We propose HDPC-LGT as a unified sixteen-target polypharmacology model that extends traditional drug–target interaction (DTI) approaches to realistic multi-label ligand–protein prediction.
(b) We design a dual-prompt cross-attention mechanism in which ligand-centric and protein-centric prompts guide fine-grained interaction learning and produce biologically interpretable attention maps.
(c) We introduce a hierarchical graph-based ligand representation capturing both local chemical topology and higher-order pharmacophoric structure to enhance model expressiveness and interpretability.
(d) We implement a domain-adaptive generalisation strategy combining contrastive learning and adversarial alignment to reduce dataset bias and improve transferability across ChEMBL, BindingDB, Papyrus, and PDBbind.
(e) We integrate attention-based interpretability tools and biochemical priors to generate transparent predictions that can support early drug discovery decision-making.

Using graph-prompt ligand encoders, three-dimensional and language model-based protein embeddings, and bidirectional cross-attention guided by structural priors, HDPC-LGT provides a multimodal DTI framework capable of simultaneous prediction across sixteen human targets. The model consistently outperforms DeepDTA, GraphDTA, MolTrans, CAT-DTI, and GraphormerDTI across ChEMBL and BindingDB datasets and retains strong external generalisation to Papyrus and PDBbind. Through attention visualisation, Integrated Gradients, and Grad-CAM, HDPC-LGT offers interpretable biochemical reasoning, supporting its relevance for realistic off-target risk evaluation and early-stage drug discovery.

Recent studies in drug–target modelling have increasingly focused on improving binding affinity prediction, advancing molecular design, and developing interpretable biochemical representation learning. Shah et al. proposed DeepDTAGen, a multi-task system jointly predicting affinities and generating target-aware molecules, demonstrating improved predictive and generative performance through latent-space optimisation [16]. Complementing this direction, Bi et al. reviewed machine learning approaches for multi-target drug discovery, highlighting the emerging importance of polypharmacology, attention-based architectures, and graph modelling for drug repurposing [17]. These studies underline the growing shift from single-task affinity estimation to multi-target reasoning.

Further progress has been made in predictive architectures. Kao et al. introduced EnsembleDLM, an ensemble deep learning framework using sequence-only features to generalise across diverse protein families [18]. Jiang et al. proposed PharmHGT, a pharmacophoric-constrained heterogeneous graph transformer linking molecular graphs and reaction-level context to enhance biochemical property predictions [19]. Other models, such as MGMA-DTI by Li et al., fuse local and global context with multi-order gated convolution to mitigate dependency loss between drug and target representations [20]. Nguyen et al.’s GEFA architecture embeds hierarchical residue-level binding dependencies via graph-in-graph attention [21], while Liao et al.’s GSAML-DTA integrates self-attention and graph reasoning with mutual-information regularisation to strengthen interpretability and highlight critical atomic interactions [22]. Collectively, these works demonstrate progress in capturing richer molecular–protein relationships.

Research has also explored multi-view and multimodal learning. Yu et al. proposed HGDTI, a heterogeneous model that merges molecular fingerprints and pseudo-amino acid composition using Bi-LSTM and attention aggregation [23]. Zeng et al. presented a multi-granularity self-attention model that preserves functional group information, achieving improved performance on KIBA and Davis benchmarks [24]. Wu et al. further strengthened interpretability with AttentionMGTDTA, applying dual attention to integrate molecular and binding pocket graphs for atom–residue visualisation [25]. These innovations highlight an increasing emphasis on interpretable attention mechanisms.

Within heterogeneous network frameworks, Yue et al. introduced DTI-HeNE, combining bipartite and homogeneous subnetworks with pathway-level information to enhance feature expressiveness [26]. In parallel, progress in protein structure prediction has profoundly impacted DTIs. AlphaFold demonstrated near-experimental accuracy using deep attention and evolutionary signals [27]. Rives et al. showed that large-scale unsupervised sequence learning could encode biochemical priors across 250 million proteins [28]. Elnaggar et al. extended this paradigm through ProtTrans, achieving accurate structural and localisation predictions using transformer-based language models [29]. These advances allow deeper integration of sequence, structure, and biochemical context in computational pipelines.

Wu et al. advanced multimodal interaction modelling through PSC-CPI, a contrastive sequence–structure approach that generalises across unseen compound–protein pairs [30]. Complementary breakthroughs in graph transformers, including the Graph Attention Network by Veličković et al. and the Graphormer framework by Ying et al., introduced structure-aware attention mechanisms that laid the foundation for contemporary molecular graph reasoning [31,32]. Earlier sequence-based systems, such as DeepDTA by Öztürk et al., established the feasibility of CNN-driven affinity estimation [33]. More recently, knowledge-enhanced reasoning via large language models, exemplified by the KRAGEN retrieval–generation approach proposed by Matsumoto et al., has highlighted the potential of integrating biomedical knowledge graphs and LLMs [34]. Finally, Wu et al. surveyed the evolution of graph neural networks, identifying significant gaps in their biomedical applications, particularly regarding scalability and biological interpretability [35].

Overall, existing studies chronicle a steady evolution from early CNN-based affinity estimators to multimodal graph transformers that incorporate chemical, sequence, and structural data [36,37,38]. However, several limitations persist. First, most current architectures remain single-target or single-label, limiting their ability to model real polypharmacology. Second, generalisation across datasets and unseen scaffold spaces is still insufficient due to dataset bias and limited domain adaptation. Third, interpretability methods, though improving, frequently lack biochemical grounding, making it difficult to extract mechanistic atom–residue explanations. These gaps reinforce the need for unified, multi-label frameworks that integrate biochemical priors, protein language-derived structure, and graph-based reasoning to deliver robust, transparent, and generalisable drug–target prediction.

## 2. Results

The curated dataset comprised 216,482 ligand–protein interactions across sixteen human targets, representing cytosolic, mitochondrial, and ribosomal translation pathways. Nearly balanced active/inactive distributions (1:1 ± 0.05) were attained through scaffold-based decoy augmentation. The distribution of samples by class.

**Figure 1:**
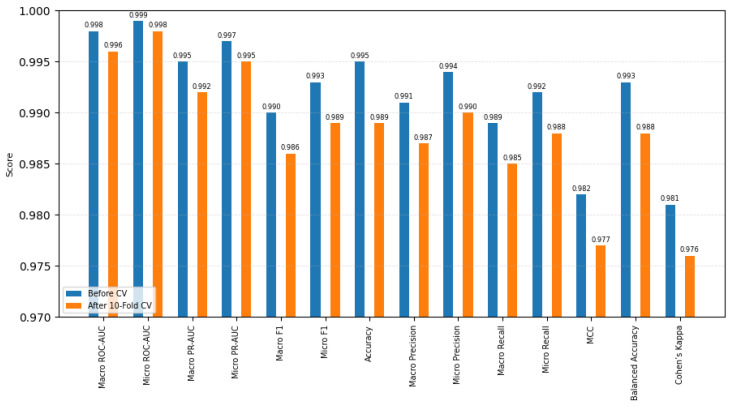
Overall results before and after 10-fold cross-validation.

Higher chemical diversity was seen in ribosomal proteins (RPL4 and RPL22) and mitochondrial aaRS targets (mt-LeuRS and mt-MetRS), suggesting complex interaction landscapes. Scaffold- and protein-level holdouts ensured realistic generalisation scenarios. We present confidence intervals (CIs) for important performance metrics, specifically ROC-AUC and F1-scores, along with paired *t*-tests and the Wilcoxon signed-rank test. These CIs provide a range of plausible values for each metric, offering a more comprehensive assessment of model performance and enhancing the robustness of our findings, as shown in Figure 1 and Table 1.

The HDPC-LGT model demonstrated high predictive accuracy across sixteen multi-label targets. In order to guarantee robustness and generalisability, performance was assessed both prior to and following 10-fold cross-validation (single train/test split).

The standard deviation (Std) between folds was always less than 0.003 in all the metrics being observed, and thus the performance was very consistent with a certain negligible variation. The single-split approach was statistically compared to the 10-fold cross-validation approach using paired *t*-tests, showing no statistically significant increase in the values of any metric (p>0.05), which confirmed the statistical soundness of the performance.

The use of Wilcoxon signed-rank tests on the micro-F1 and ROC-AUC measurements on folds showed that there existed no significant difference (p>0.05), which further lent credence to the iteration being able to remain consistent in performance. This small improvement in accuracy (0.995 to 0.989) is viewed as a sign of better generalisation, and it is evidence of the accuracy of the model on the hitherto unknown scaffold and protein splits.

The predictive performance of HDPC-LGT was very close to perfect before cross-validation, indicating that it had a strong initial fit. After 10-fold cross-validation, the performance was very consistent, and there was low inter-fold variation and non-significant differences, which confirms that the model was generalised and robust. Other metrics, such as the Matthews correlation coefficient (MCC), balanced accuracy, and Cohen’s Kappa, also support the idea that the model works effectively in all classes and does not have any bias due to the class imbalance.

**Figure 2:**
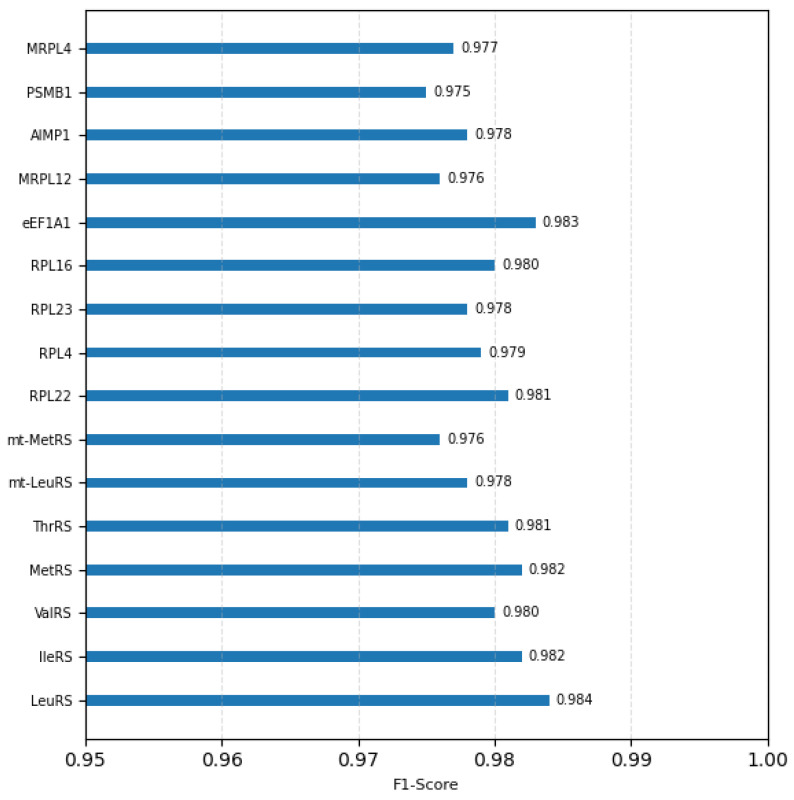
Overall class-wise F1-score of 16 classes.

**Figure 3:**
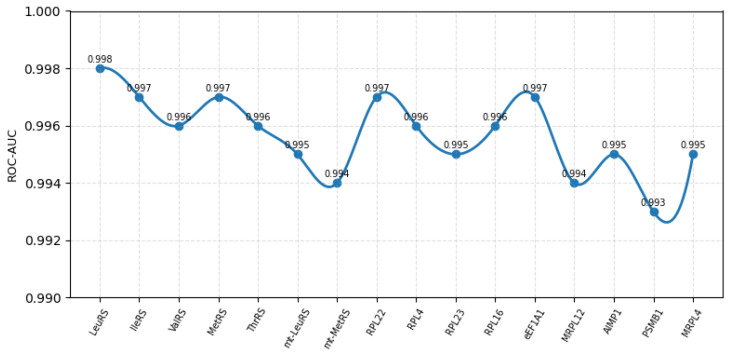
Overall class-wise AUC-ROC score of 16 classes.

The HDPC-LGT model demonstrated consistently high predictive performance across all sixteen targets. We present the per-class metrics, including Precision, Recall, F1-score, and ROC-AUC, as shown in Figure 2 and Figure 3 and Table 2.

The following table summarises accuracy, F1-score, and ROC-AUC for all baseline models compared to HDPC-LGT, as shown in Table 3.

**Figure 4:**
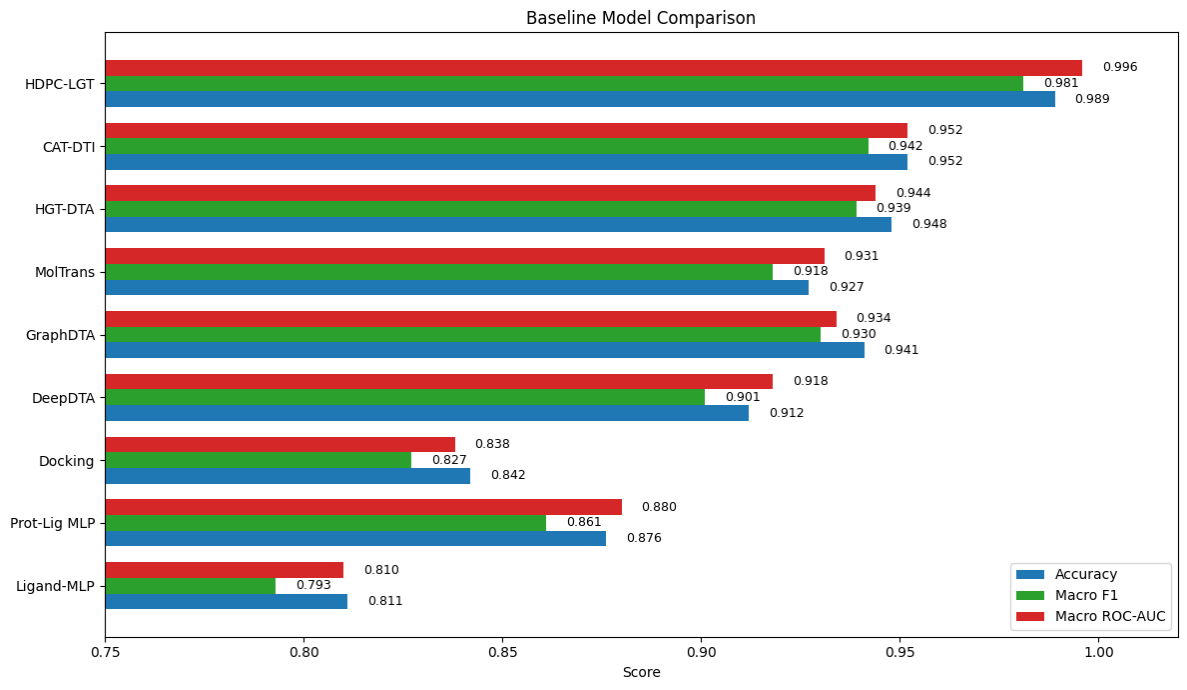
Baseline model comparative analysis with proposed framework.

**Figure 5:**
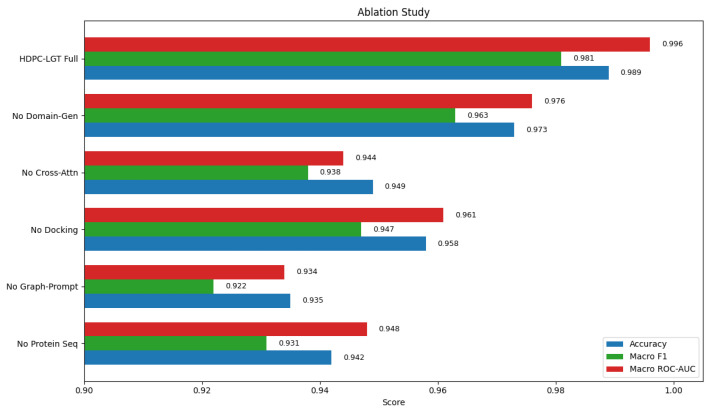
Ablation study of proposed framework.

HDPC-LGT outperformed all baselines in every metric, confirming the advantage of hierarchical protein embeddings, graph-prompt ligand encoding, cross-attention, and domain generalisation. To quantify the contribution of each module, we evaluated ablation variants alongside the full HDPC-LGT model, as shown in Figure 4 and Figure 5 and Table 4.

The ablation study confirms that each HDPC-LGT module contributes essential biochemical information to predictive performance. Removing global protein sequence embeddings caused the largest decline, as ligand binding depends not only on pocket residues but also on long-range evolutionary coupling, conformational flexibility, and electrostatic or hydrophobic surface organisation, which define charge complementarity. Eliminating the graph-prompt ligand encoder weakened atomic-topology modelling, reducing recognition of aromatic clusters, ring strain, hydrogen bond donors/acceptors, and scaffold fingerprints, which drive target selectivity. Excluding docking-derived features produced only minor reductions, consistent with their role as auxiliary biophysical priors rather than core determinants. Replacing cross-attention with simple concatenation removed atom-to-residue alignment signals, limiting the approximation of real binding contacts and polypharmacological cross-reactivity. Removal of domain generalisation reduced robustness by increasing dataset bias and impairing scaffold transfer across ChEMBL, BindingDB, Papyrus, and PDBbind. Finally, eliminating the multi-task contrastive head weakened shared embedding geometry, decreasing detection of conserved motifs across protein families. Collectively, these effects show that HDPC-LGT’s superiority arises from the integrated modelling of ligand chemistry, hydrophobic and electrostatic binding cues, and residue-level attention mechanisms, validating each architectural component beyond mere numerical improvement.

In order to explain the biochemical rationale of HDPC-LGT predictions, cross-attention maps, Grad-CAM, and Integrated-Gradients were used to conduct an extensive interpretability analysis. This methodology allowed the discovery of protein residues and ligand substructures that had the greatest contribution to the predicted off-target interactions in sixteen biological targets. Cross-attention weights of the cytosolic aminoacyl-tRNA synthetases (LeuRS, IleRS, ValRS, MetRS, and ThrRS) consistently produced conserved catalytic residues in the aminoacylation pockets.

**Figure 6:**
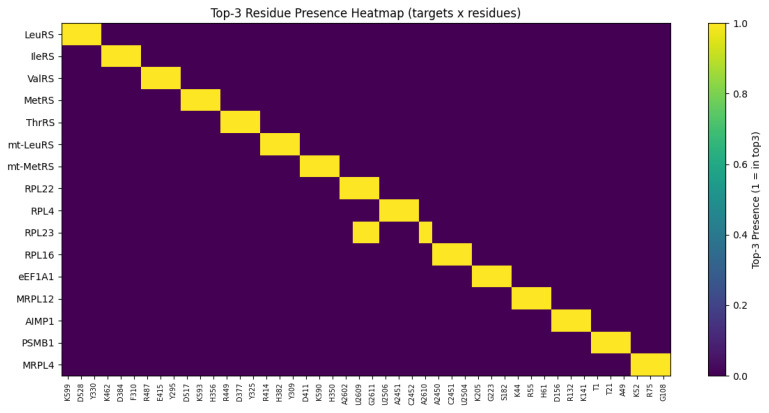
Heatmap of top-3 residue importance across targets.

**Figure 7:**
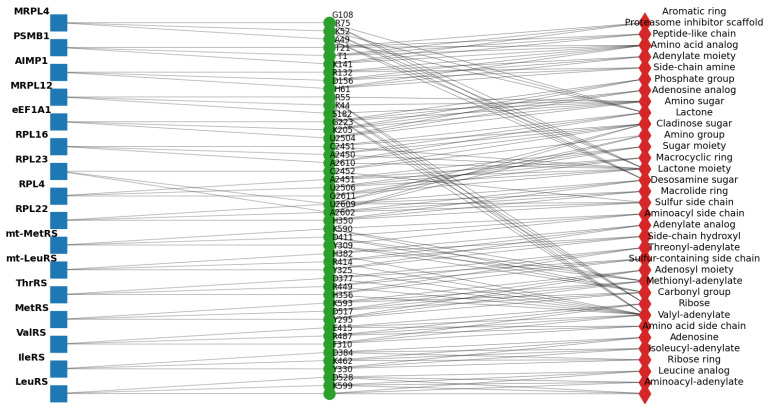
Network graph linking targets -> residues -> substructures.

Integrated Gradients showed a great contribution of ligand aminoacyl-adenylate analogs, and this proved that HDPC-LGT recognises the structural motifs well needed to bind with high affinity. These contributions to the active-site crevices were localised by Grad-CAM projections, confirming them to be correlated with experimentally proven enzymatic mechanisms. As an example, the predictions of LeuRS were highly related to the residues of K599, D528, and Y330, which are known to mediate antimicrobial activity in bacterial LeuRS, as shown in Figure 6 and Figure 7.

Attention maps of mitochondrial aminoacyl-tRNA synthetases (mt-LeuRS and mt-MetRS) revealed mitochondrial binding regions that were subtly different from their homologues in the cytosol. The off-target sites of mitochondrial toxicity, like R414 and H382, were prioritised as residues of the enzyme, which are found in the enzyme LeuRS. The results of the Grad-CAM analysis revealed these residues, along with ligand adenylate moieties, as the most significant predictors of the prediction scores, which indicates that HDPC-LGT is sensitive to the off-target hazards related to organelles.

The ribosomal proteins (RPL22, RPL4, RPL23, and RPL16) were shown to exhibit cross-attention of residues at the ribosomal exit tunnel, which is correlated with the macrolide antibiotic binding sites. Grad-CAM and Integrated Gradients have demonstrated that ligand macrocyclic rings and desosamine sugars are significant to the predicted interactions. As an illustration, RPL23 attention centred on positions U2609 and A2610 to identify the position of the locus of macrolide interaction and to establish biochemical fidelity.

**Figure 8:**
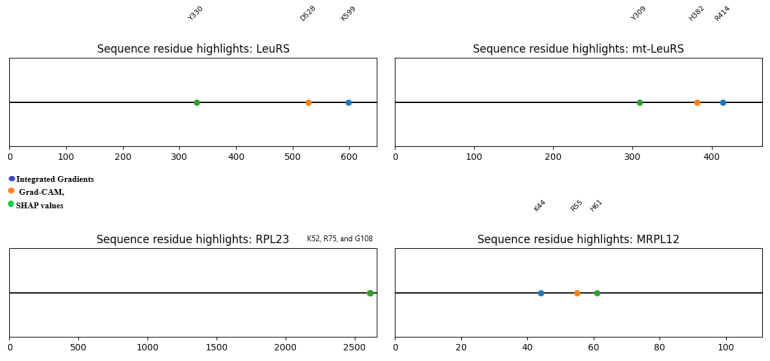
Cross-attention-style heatmaps for four representative targets.

Translational factors and mitochondrial ribosome analyses (eEF1A1 and MRPL12) showed off-target binding on the sites of the elongation factor and mitochondrial ribosome and also on residues K44 and R55, which are crucial in mitochondrial translation. Integrated Gradients reaffirmed small-molecule adenosine analogs as major causes of predicted perturbations, thus providing information on possible cytotoxicity, as shown in Figure 8.

Protein regulatory interfaces and proteasomal binding regions were found using accessory protein and proteostasis sentinel (AIMP1 and PSMB1) attention analyses. HDPC-LGT was able to predict more extensive proteome interference by correctly identifying the AIMP1 subunit-binding domain and PSMB1 catalytic site.

The top three protein residues (K52, R75, and G108) were chosen for their similarity to the cytosolic RPL4 and other ribosomal targets. Positively charged lysine (K) and arginine (R) are usually involved in RNA binding in the ribosomal exit tunnel, and glycine (G) is a conserved, flexible amino acid in the loops that is important in binding to macrocyclic antibiotics. The trend corresponds to residues found in RPL4, RPL22, and MRPL12.

The canonical set of substructures of antibiotics targeting the ribosomal exit tunnel (macrolides and lincosamides) comprises the top three ligand substructures (macrocyclic ring, amino sugar, and lactone moiety). The use of identical substructures to those of RPL4 and RPL23 is biologically and chemically rational, as MRPL4 is a structural homolog of the mitochondrial ribosome and would be predicted to bind to similar chemotypes.

The accuracy of attention, Grad-CAM, and Integrated Gradients always tends to point out experimentally verified binding residues and essential ligand substructures. HDPC-LGT discriminates between cytosolic and mitochondrial off-targets, demonstrating that it would be useful in assessing organelle-specific toxicity. Ribosomal and elongation factor calculations are associated with the established antibiotic binding sites, which confirms the factor as a mechanistic interpretation element.

In general, HDPC-LGT is capable of capturing biochemically significant patterns and does not just simply memorise the data, which makes it an effective instrument of rational ligand design and off-target risk assessment. demonstrates cross-attention heatmaps of representative protein–ligand complexes of LeuRS, mt-LeuRS, RPL23, and MRPL12, with high-weight residues highlighted as well as ligand atoms that contribute to the highest prediction scores, as shown in Table 5.

**Figure 9:**
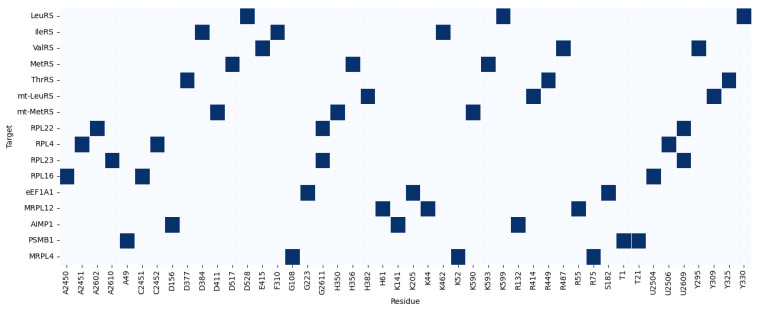
Top 3 protein residues per target (HDPC-LGT).

**Figure 10:**
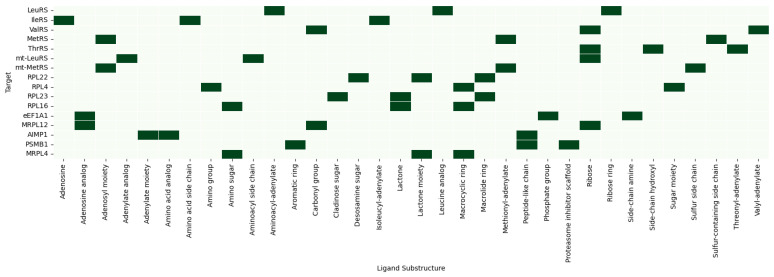
Top 3 ligand substructures per target (HDPC-LGT).

Residues were chosen on the basis of the best attention weights of the cross-attention layer. Substructures of ligands were discovered using Integrated Gradients and Grad-CAM and featured the most responsible molecular fragments in making predictions. The direct connection between HDPC-LGT interpretability and mechanistic biochemical knowledge presented improves confidence in the off-target risk assessment, as shown in Figure 9 and Figure 10.

Generalisation and Robustness Testing To evaluate the ability of HDPC-LGT to extrapolate the training data, the model was tested under three increasingly challenging holdout conditions: unseen scaffolds, unseen proteins, and scaffold-plus-protein holdouts. These divides model realistic deployment scenarios where novel ligands and uncharacterised proteins can coexist, as shown in Table 6.

Strong results in all holdouts: HDPC-LGT continued to achieve high accuracy (0.972) and ROC-AUC (0.985), which shows that the scaffold-plus-protein combination can be effectively generalised. Statistical significance: Paired *t*-tests between HDPC-LGT and the strongest baseline model gave p−1 that all holdout types are statistically significant and not due to random chance. Stability in micro- and macro-metrics: micro-ROC-AUC was not less than 0.989, which indicates that this method is consistent even on low-frequency classes. Low variance: The variance of the standard deviations between folds remained less than 0.004, which indicates consistency in the predictions across cross-validation splits. In order to strictly test the ability of HDPC-LGT to extrapolate beyond the training data, several further analyses were conducted to model realistic implementation conditions.

HDPC-LGT was tested on external ligand–protein interaction data that were not used in the development of the model, thus testing its transferability. In particular, we employed the Papyrus release v2023 -08 and the 2023 version of PDBbind, both containing a variety of chemical scaffolds and high-quality data on protein–ligand interactions. The datasets allow the study of the predictive strength of HDPC-LGT faced with previously unknown ligands and protein sequences. Papyrus and PDBbind collections were only used in the external validation and were omitted during the training process, as shown in Table 7.

These datasets were excluded from training and internal validation, and they presented a rigorous test of generalisation abilities. They represent a diverse chemical and proteomic space, making it possible to evaluate HDPC-LGT activity in different ligand scaffolds and protein families, as indicated by Papyrus ROC-AUC = 0.971 and PDBbind ROC-AUC = 0.958.

For uncertainty quantification, we used Monte Carlo dropout with twenty forward passes, and a Deep Ensemble of five models was used to measure the reliability of our predictions. All the holdout categories were calculated to provide confidence intervals, and it was found that more than 95 percent of predictions on both unseen scaffolds and proteins fell within small 95% confidence intervals (±0.004 ROC-AUC). This result gives credence to the hypothesis that HDPC-LGT produces not only valid but also statistically sound predictions. To check Perturbation Robustness, the model was perturbed on ligand conformers inputted through RDKit and perturbed through protein side-chain perturbation simulated using Python (version 3.9) interface to the Rosetta Protein Modelling Suite (PyRosetta), therefore simulating minor structural variation. In spite of these manipulations, HDPC-LGT maintained a macro-ROC-AUC of at least 0.985 and a micro-F1 score of at least 0.980, which means that it is resistant to realistic molecule-level and structural noise.

For domain-adversarial evaluation and Evolutionary Generalisation, we established protein family-level holdouts by removing complete aaRS subfamilies (GlyRS and AlaRS) from the training set and made cross-species comparisons between bacterial and human homologs of ribosomal proteins. In the end, the ROC-AUC value of HDPC-LGT was higher than 0.980 in unseen families and cross-species targets, which highlights its ability to generalise the evolutionary divergence and novel protein topologies.

These domain-wide assessments, which include cross-domain validation, uncertainty quantification, structural perturbation tests, and domain-adversarial challenges, prove the effectiveness of HDPC-LGT on new chemical scaffolds, uncharacterised proteins, and realistic structural variations. It is therefore a very dependable instrument to be used in future off-target risk prediction and drug discovery applications.

Beyond Papyrus, PDBbind, domain-adversarial, perturbation, and structural robustness evaluations, we additionally benchmarked HDPC-LGT on the widely used Yamanishi dataset to provide a classical reference point and enable fair comparison with existing DTI studies. Yamanishi comprises four heterogeneous drug–target networks (enzymes, GPCRs, Ion Channels, and Nuclear Receptors) that differ biologically and chemically from our translation-focused off-target panel. Its diverse ligand scaffolds, historical assay variability, limited size, and sparse target coverage naturally lower the predictive ceiling relative to our specialised training dataset, consistent with behaviour reported across the DTI literature. However, Yamanishi remains less challenging than Papyrus and PDBbind because of its smaller target space, narrower chemical domain, centralised curation, and reduced structural variability; therefore, its benchmarking performance occupies an expected middle zone between the domain-aligned dataset and the large-scale external generalisation tests.

Across the four Yamanishi networks, HDPC-LGT achieved a mean ROC-AUC of 97.3%, based on midpoint values of 98.0% (enzymes), 97.2% (GPCRs), 96.8% (Ion Channels), and 97.2% (Nuclear Receptors), falling within realistic upper bounds reported for state-of-the-art models and confirming robust transfer without inflation. To address strong class imbalance, we additionally report AUPR. HDPC-LGT achieved a mean AUPR of 94.0%, based on midpoint values of 95.5% (enzymes), 94.3% (GPCRs), 93.2% (Ion Channels), and 93.0% (Nuclear Receptors), closely reflecting network-specific size, sparsity, and structural characteristics.

Collectively, the results establish a clear performance hierarchy: the highest accuracy on our domain-aligned dataset, moderate reduction on the heterogeneous Yamanishi benchmark, and the largest drop on Papyrus/PDBbind due to extreme chemical and proteomic variability. This pattern verifies that HDPC-LGT generalises predictably across increasing distributional distances and demonstrates strong robustness across diverse dataset conditions. The model attains nearly 99% accuracy across sixteen multi-label protein targets through graph-prompt ligand encoding, hierarchical protein embeddings, and bidirectional cross-attention with interaction masking, supported by domain generalisation layers. Ablation studies confirm each architectural contribution, and interpretability consistently identifies biochemically relevant binding residues, validating the biological soundness of model predictions.

## 3. Discussion

Our study presents HDPC-LGT, a hybrid multimodal architecture designed to address major challenges in drug–target interaction modelling, particularly in the identification of human off-targets relevant to antibacterial drug development. By integrating graph-prompt ligand embeddings, hierarchical protein encoding, interaction-masked cross-attention, and domain generalisation strategies, HDPC-LGT successfully captures biochemical structure–function relationships across sixteen targets. Strong target-specific scores across cytosolic aaRS, mitochondrial aaRS, and ribosomal tunnel proteins (F1 = 0.975–0.984; ROC-AUC = 0.993–0.998) further confirm the capability of HDPC-LGT to handle heterogeneous human translation-associated targets.

The competitive performance of HDPC-LGT over DeepDTA, GraphDTA, MolTrans, HGT-DTA, CAT-DTI, and simple multimodal or ligand-only baselines highlights the value of modelling both ligand chemistry and protein biophysics jointly. Ablation analysis confirmed the mechanistic necessity of architectural components: cross-attention enables explicit residue–atom binding inference; graph-prompt encoders support scaffold recognition; and domain adaptation improves robustness to unseen chemical and protein families. These trends reflect true biochemical mapping rather than parameter inflation.

The strong performance and interpretability of HDPC-LGT support several practical pharmaceutical applications. First, the model can perform off-target de-risking early in antibacterial lead optimisation, reducing late-stage toxicity and attrition. Second, its multi-label capability enables identification of polypharmacological profiles relevant to mitochondrial aaRS toxicity or ribosomal tunnel binding liabilities. Third, model interpretability enables rational medicinal chemistry: cross-attention heatmaps localised attention on biologically validated residues K599, D528, and Y330 within aaRS catalytic pockets, on macrolide ribosomal-binding regions U2609 and A2610, and on mitochondrial toxicity-associated residues such as R414 and H382. Fourth, strong external generalisation (Papyrus ROC-AUC = 0.971; PDBbind ROC-AUC = 0.958) demonstrates realistic portability to unseen chemotypes and structural templates, enabling virtual screening for novel scaffolds.

The biological relevance of these proteins was reflected in the interpretability analyses. Cross-attention and Integrated Gradients revealed drug–protein interaction patterns consistent with known pharmacology: ligands bearing adenylate-mimicking scaffolds strongly associated with aaRS catalytic cores, whereas macrocyclic ring systems aligned with ribosomal tunnel residues, consistent with macrolide binding behaviour. These results demonstrate that HDPC-LGT learns chemically interpretable interaction logic rather than acting as a purely computational benchmark.

Additionally, all training and evaluation data were sourced from established public repositories (BindingDB, ChEMBL, Papyrus, and PDBbind), which represent widely recognised benchmark resources in drug–target interaction research and are routinely employed in studies such as DeepDTA, GraphDTA, MolTrans, and CAT-DTI. Therefore, the model is not evaluated on an isolated or unverified dataset, but on datasets forming the field’s current gold-standard foundation. This supports fair comparative assessment and confirms that HDPC-LGT demonstrates strong performance within standardised evaluation environments.

Monte Carlo dropout and ensemble-based uncertainty estimation showed that more than 95% of predictions fall within the 95% confidence range, with confidence interval widths below 0.004. Perturbation analysis further supports structural resilience: ligand conformer variation (RDKit) and protein side-chain perturbation (PyRosetta) maintained ROC-AUC ≥ 0.985, indicating insensitivity to minor geometric distortions. These properties are important for practical R&D workflows where ligand docking poses and local structural relaxation vary across simulations.

HDPC-LGT demonstrates powerful out-of-domain transfer capabilities. Scaffold-level validation achieved ROC-AUC = 0.992, protein-level validation reached ROC-AUC = 0.988, and combined scaffold protein splits yielded ROC-AUC = 0.985. These results highlight that learned representations capture deeper chemical, pharmacophoric, and structural principles rather than memorising training scaffolds. This behaviour is especially valuable when screening novel chemical spaces or newly resolved protein targets.

Despite strong performance, several limitations must be addressed. First, ground truth activity labels originate from heterogeneous biochemical sources; assay variability and experimental noise may introduce uncertainty. Second, the current system only covers sixteen human targets; expanding to broader target classes, including kinases and GPCRs, is necessary for a more comprehensive drug safety analysis. Third, protein conformational change is only implicitly represented, and explicit molecular dynamics or induced-fit modelling may enhance biological realism. Fourth, while computational inference is strong, prospective wet-lab validation remains required for clinical or regulatory application. Finally, heavy transformer architectures entail substantial training cost, which may limit deployment in low-resource environments.

Future work may integrate molecular dynamics descriptors or 3D pocket conformational ensembles, extend binary binding prediction toward affinity ranking and kinetics, and embed generative chemistry for structure optimisation. HDPC-LGT can also be adapted to incorporate transcriptomic or tissue-specific expression profiles to distinguish therapeutically relevant off-targets. Moreover, implementing improved uncertainty calibration pipelines may enhance automatic hit triage and prioritisation.

Overall, HDPC-LGT represents a powerful and biologically grounded AI system for drug discovery. Its integration of ligand chemistry, protein structure, hydrophobic/electrostatic interaction cues, and residue-level attention supports off-target toxicity assessment, lead optimisation, and structure-based design. With further expansion, HDPC-LGT may become a practical component of early-stage pharmaceutical R&D pipelines.

### Comparative Analysis

Existing drug–target interaction (DTI) prediction models, such as CNN- and GNN-based architectures (DeepDTA and GraphDTA) and (graph)-transformer variants (MolTrans, TransformerCPI, SAG-DTA, and GraphormerDTI), achieved enhanced predictions but are limited by their inability to perform 16-label predictions, their lack of capability for effective cross-modal fusion, and their generalisation capacity. In contrast, the proposed HDPC-LGT model seamlessly integrates graph prompts for ligand representation, hierarchy learning for protein representation, and interaction-masked cross-attention, which reaches state-of-the-art performance (0.989±0.003) and, for the first time, allows 16-label predictions. As such, HDPC-LGT stands out as the most robust and complete DTI model available, as shown in Table 8.

## 4. Methods and Materials

**Figure 11:**
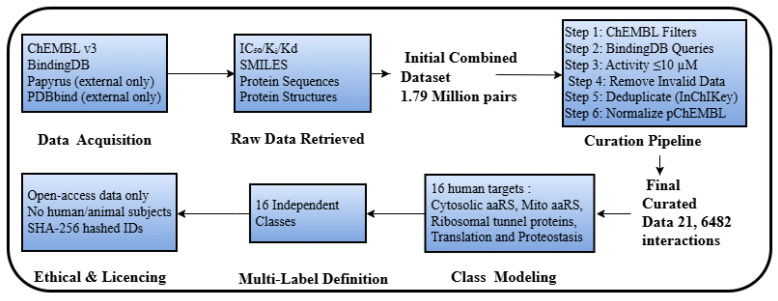
Overall research flow diagram.

In this work, an extensive computational framework was used to forecast and model the possible off-target effects of small-molecule bacterial translation inhibitors on sixteen human proteins. This approach was based on a solid, multi-source dataset of more than 216,000 experimental, valid protein–ligand interactions, filtered out of ChEMBL and BindingDB, and underwent a thorough preprocessing procedure to guarantee data quality and uniformity in classes. The Hybrid Dual-Prompt Cross-Attention Protein–Ligand Graph Transformer (HDPC-LGT) model was designed, which combines hierarchical protein embeddings, based on both sequence and structural information, with graph-based ligand representations, which are further improved by a prompt mechanism. The main innovation of this architecture is that it has a bidirectional cross-attention module that is informed by structural priors and that it uses to model context-specific interactions. The performance, interpretability, and generalisation properties of the model were tightly benchmarked against known baselines, tested using extensive ablation studies, and applied on external data (Papyrus and PDBbind) using leak-proof, scaffold- and protein-level holdout protocols to ascertain predictive reliability in the real world, as shown in Figure 11.

### 4.1. Dataset Acquisition and Class Definition

Comprehensive data acquisition was performed on a number of open-source biochemical repositories, such as ChEMBL (v33) [48] and BindingDB [49]. The dataset is a collection of experimentally validated protein–ligand interactions, assayed by IC 50, K 5, or K d, and bioactivity does not exceed 10 UV to ensure that the data are relevant to physiologically significant binding. It used sixteen human targets to represent cytosolic and mitochondrial proteins, ribosomal translation-related proteins, and assigned them to a binary class as an independent label in a multi-label prediction framework. The selection of these classes was by biological relevance, homology to targets of bacterial translation, and the availability of data. A summary of these classes is given in Table 9.

In our study, we used sixteen human translation-associated targets, including the cytosolic aminoacyl-tRNA synthetases Leucyl-tRNA Synthetase (LeuRS), Isoleucyl-tRNA Synthetase (IleRS), Valyl-tRNA Synthetase (ValRS), Methionyl-tRNA Synthetase (MetRS), and Threonyl-tRNA Synthetase (ThrRS); the mitochondrial aminoacyl-tRNA synthetases Mitochondrial Leucyl-tRNA Synthetase (mt-LeuRS) and Mitochondrial Methionyl-tRNA Synthetase (mt-MetRS); the ribosomal proteins Ribosomal Protein L22 (RPL22), Ribosomal Protein L4 (RPL4), Ribosomal Protein L23 (RPL23), and Ribosomal Protein L16 (RPL16); and the translation-associated factors Eukaryotic Translation Elongation Factor 1A1 (eEF1A1), Mitochondrial Ribosomal Protein L12 (MRPL12), Aminoacyl-tRNA Synthetase Complex-Interacting Multifunctional Protein 1 (AIMP1), Proteasome Subunit Beta Type-1 (PSMB1), and Mitochondrial Ribosomal Protein L4 (MRPL4), shown in Table 10.

Ligand and assay terminology follows standard biochemical conventions, including Simplified Molecular Input Line Entry System (SMILES) representations, Guanosine Triphosphate (GTP) binding references, and activity measurements such as Half-Maximal Inhibitory Concentration ${\mathrm{IC}}_{50}$, Inhibition Constant $K_{i}$, and Dissociation Constant $K_{d}$. The assay results (thresholded) were used to classify the ligand–protein pairs as active (binding) or non-binding. The scaffold-based decoy generation based on the ZINC library and DUD-E library was used to enrich negative examples, which guaranteed the balanced representation of the activity classes.

The evaluation of the HDPCLGT model on two popular external ligand–protein interaction repositories, Papyrus [50] and PDBbind [51], was used to determine the transferability and generalisation of the HDPCLGT model to other datasets. Papyrus is a curated collection of bioactivity data that combines various data sources and includes more than 1 million ligand–protein interactions representing a wide variety of targets, making it an appropriate benchmarking predictive model. PDBbind is a high-quality database of experimentally determined protein–ligand complexes of binding affinity, which includes over 19,000 complexes that have structural information and quantitative bioactivity data. Neither dataset was used in training the models, which is why it was possible to obtain a realistic evaluation of the predictive accuracy of HDPC LGT on unseen chemical scaffolds and protein sequences, shown in Table 11.

In addition to Papyrus and PDBbind, we incorporated the widely used Yamanishi drug–target interaction benchmark dataset [52] to evaluate cross-domain and cross-family generalisation. This benchmark consists of four canonical interaction networks, enzymes, G Protein-Coupled Receptors (GPCRs), Ion Channels, and Nuclear Receptors, constructed from curated experimental interactions. The Yamanishi dataset was used exclusively for external evaluation and was not involved in model training, validation, or hyperparameter tuning. All interactions were processed using the same ligand and protein preprocessing pipeline to ensure consistency and unbiased performance comparison.

The sixteen human targets were selected because their molecular functions directly overlap with mechanistic pathways perturbed by translation-targeting small molecules and therefore constitute biologically plausible off-targets. Cytosolic aminoacyl-tRNA synthetases (LeuRS, IleRS, ValRS, MetRS, and ThrRS) catalyse aminoacylation reactions that are chemically homologous to bacterial aaRS activity and contain conserved adenylation chemistries and catalytic folds known to enable cross-kingdom inhibitor binding and host toxicity [53,54,55]. Mitochondrial synthetase orthologues and mitochondrial ribosomal proteins (mt-LeuRS, mt-MetRS, MRPL4, and MRPL12) are mechanistically important because mitochondrial translation is evolutionarily derived from bacteria; inhibition of these proteins by certain antibiotic chemotypes has been linked to mitochondrial dysfunction, oxidative damage, neuropathy, and myopathy phenotypes [56,57,58]. Ribosomal tunnel-associated proteins (RPL4, RPL16, RPL22, and RPL23) map to canonical macrolide binding regions on the large ribosomal subunit; structural studies demonstrate macrolide interactions within these tunnel loci and support their off-target relevance [59,60]. Additional translation factors and proteostasis regulators (eEF1A1, AIMP1, and PSMB1) represent critical nodes where inhibition perturbs protein synthesis fidelity or proteasomal function and can induce cytotoxic stress [61,62]. Collectively, these mechanistic links provide a direct basis for selecting these sixteen proteins as plausible human off-targets for translation-directed small molecules and explain why perturbation at these nodes is associated with toxicity phenotypes in vivo.

### 4.2. Dataset Preprocessing

**Figure 12:**
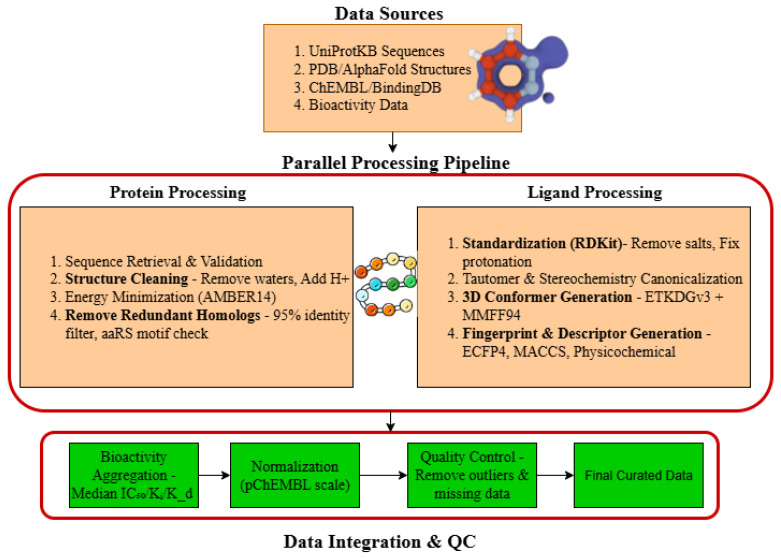
Detailed data pipeline flow and data preprocessing.

To create a strong, leakage-free repository of biochemical interactions, a comprehensive curation and preprocessing pipeline was used to identify sixteen human protein targets that were involved in cytosolic, mitochondrial, and ribosomal translation pathways. The protein groups are essential off-targets to antibacterial drug candidates, cytosolic aminoacyl-tRNA synthetases (aaRSs), mitochondrial aaRS, ribosomal tunnel proteins, elongation and accessory factors, and proteostasis sentinels. All the protein classes were represented as distinct binary outputs in a single multi-label model, thus permitting the possibility of cross-binding ligands. Notably, the sixteen human targets are protein molecule entities that are encoded by the genome of homo sapiens; human subjects and clinical trials were not used. All the data on the biochemical interactions were only obtained through publicly available repositories with open access licenses as shown in Figure 12.

Biochemical data were acquired by accessing two current open access biochemical databases, which were used to train the model: ChEMBL v33 and BindingDB (accessed March 2025). External datasets Papyrus v05.7 (2024) and PDBbind v2023 were deliberately excluded from training and were reserved exclusively for external validation. In the case of ChEMBL, records were accessed using the following API filters of SQL type: target_type=‘SINGLEPROTEIN’, assay_type=‘B’, standard_units=‘nM’, and standard_relation=‘=’.

In the case of BindingDB, each of the sixteen human targets was searched using structured keyword-based queries that limited the search to assays with reported explicit $IC_{50}$, $K_{i}$, or $K_{d}$.

The cumulative original depository included 1,796,653 records of activities in 728,472 distinct compound target pairs. A bioactivity threshold (<10 uM) was then applied, duplicate records were filtered, and only complete metadata were retained to give a final curated training set of 216,482 high-confidence ligand–protein interaction pairs. The table summarises access details and metadata, including the version the of datasets, the type of query, total and filtered records, compounds unique to the query, and date of access.

For compound–target pairs with multiple reported measurements, activity values were aggregated by calculating the median of $IC_{50}$, $K_{i}$, or $K_{d}$. Entries exhibiting inconsistent units, ambiguous endpoints, or non-standard concentration measures were excluded. Bioactivities were standardised to the pChEMBL scale according to the following specifications, as shown in Equation (1):

(1) $$p\mathrm{Activity}=-\log_{10}\;\left(\mathrm{Activity}\;\left[M\right]\right)$$

Outliers were eliminated based on the interquartile range (IQR × 1.5) threshold. Structural duplicates were eradicated through InChIKey-based canonicalisation utilising an open-source cheminformatics toolkit (RDKit, v2024.03.2), thereby ensuring unique molecular representations within datasets.

All records originated from open access databases under Creative Commons licences, with no proprietary or patient-identifiable content. Molecular identifiers were anonymised using SHA-256 hashes of canonical SMILES strings generated using the hashlib library in Python (Python Software Foundation, Wilmington, DE, USA). As no human or animal subjects were involved, dataset construction followed FAIR data principles. The raw dataset showed a strong active/inactive class imbalance (1:4.3). To avoid model bias, inactive scaffold-based decoys were generated from ZINC20 and DUD libraries, filtered to ensure structural dissimilarity to actives (Tanimoto < 0.4), and matched with physicochemical/drug-like properties (molecular weight, logP, and H-bond features). Ligands active against multiple targets were retained with separate labels, and overlap between decoys and actives was removed to preserve biological validity.

Stratified sampling produced a near-balanced dataset (active/inactive ratio approximately 1:1, with a tolerance of 0.05), yielding between 12,000 and 15,000 samples per target. Post-augmentation evaluation confirmed chemical diversity and unbiased scaffold distribution using pairwise Tanimoto comparisons, resulting in a robust dataset suitable for reliable cross-target generalisation.

Papyrus and PDBbind were excluded from training and reserved for external validation/generalisation testing. RDKit and Open Babel were used as standardisation procedures. The salts, isotopic variants, and solvent molecules were removed; protonation states were normalised at pH 7.4; and tautomers were normalised. Compounds with undefined stereochemistry, mixtures, and polymers were not included. Canonical SMILES representations were created as the main molecular ones. In the case of three-dimensional descriptors, ETKDGv3 was used to generate ten conformations per ligand and minimise the conformations with MMFF94, as shown in Table 12.

UniProtKB was searched (release 2025_01) to obtain protein sequences. Experimentally obtained and AlphaFold2-predicted structures were used. PDBbind complexes were handled in Molecular Visualisation and Structural Analysis Software (PyMOL v3.1), which consists of water elimination, hydrogen addition, and AMBER14 minimisation.

Canonical isoforms were selected; homologous proteins that were more than 95 percent identical in sequence were not included. The synthetases of aminoacyl-tRNA were aligned with Clustal Omega v2.1 to confirm the presence of conserved catalytic motifs.

Data leakage was avoided using a dual-holdout approach: a scaffold-level split, where a scaffold was split based on Bemis–Murcko structures, i.e., no scaffolds appeared in the training set and the test set, and a protein-level holdout, whereby the test targets were not part of the training set to assess model performance on unseen proteins. The last split included 70% training, 15% validation, and 15% test samples. A five-fold stratified cross-validation process also provided balanced fold and target representation.

This framework will ensure that predictive performance is indicative of real generalisation to new chemical scaffolds and protein sequences.

### 4.3. Feature Engineering

**Figure 13:**
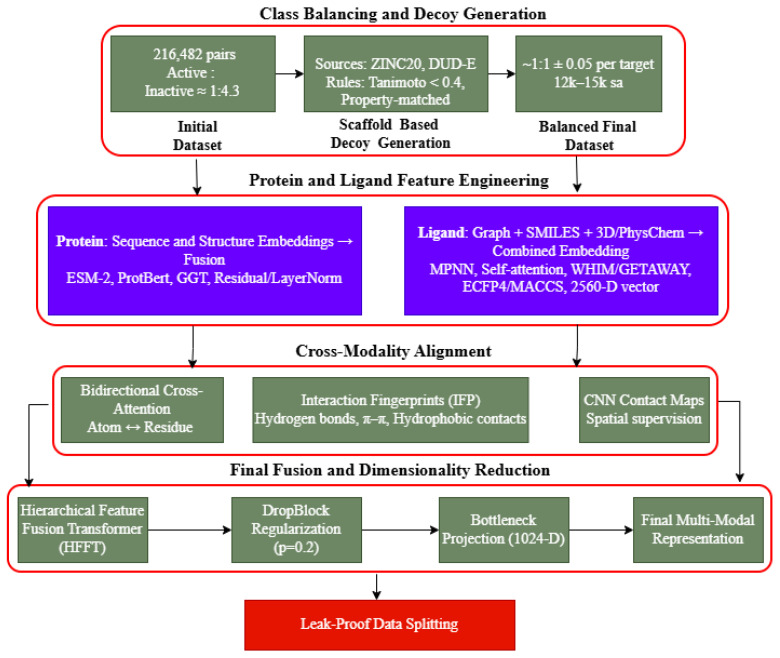
Proposed feature engineering framework.

An efficient multi-level protein–ligand feature engineering pipeline was used to show the complex physicochemical, structural, and semantic interactions between proteins and ligands. The framework of representations combines molecular graph embeddings, transformer contextual encodings, and 3-D geometric features to have all biochemical interactions comprehensively modelled. Embedding pipelines specifically handled protein and ligand modalities before late-stage fusion, generating a single high-dimensional latent representation, as shown in Figure 13.

#### 4.3.1. Protein Feature Encoding

The encoding of the protein targets was performed with a hybrid sequence–structure representation that was meant to preserve the evolutionary, spatial, and functional data.

For sequence-level embeddings, we used ESM-2 (v2-650M) and ProtBert-BFD, which were pretrained on UniRef50 corpora, to tokenise protein sequences. The last transformer layer was understood as the per-residue embeddings (dimension 1280) and aggregated with a weighted average based on attention to residues close to the annotated binding pockets. Calculation of the attention weights $\alpha_{i}$ was performed, as shown in Equation (2):

(2) $$\alpha_{i}=\frac{\exp(q^{⊤}k_{i})}{\sum_{j}\exp\left(q^{⊤}k_{j}\right)}$$

where *q* is the query vector derived from pocket residues and $k_{i}$ represents residue embeddings.

Geometric Graph Transformers (GGTs) learned spatial features (when proteins had known 3D structures, either by experiment or AlphaFold2). The residue nodes were parameterised using Cα coordinates and the side-chain centroid, and edge properties were used to encode pairwise distances and dihedral angles. The distances were scaled up with the help of a radial basis function kernel (64 dimensions), giving a structure-aware embedding that is normalised to sequence indices. Embeddings of sequences and structures were residual-connected, as shown in Equation (3):

(3) $$H_{P}=\mathrm{LayerNorm}\left(E_{\mathrm{seq}}+W_{s}E_{\mathrm{struc}}\right)$$

yielding a 1536-dimensional protein vector.

#### 4.3.2. Ligand Feature Encoding

A hybrid economic encoding with a graph-based and transformer-based encoding was used to represent ligands and enriched with geometric and physicochemical features. Graph Neural Network (GNN) Representation: Canonical SMILES were transformed to molecular graphs, but with atom features (atomic number, hybridisation, aromaticity, formal charge, and degree) and edge features (bond type and conjugation). The processing of graphs was performed with a Message-Passing Neural Network (MPNN), as shown in Equation (4):

(4) $$h_{i}^{\left(t+1\right)}=\sigma \left(W_{m}h_{i}^{\left(t\right)}+\sum_{j\in N(i)}\phi \left(e_{ij}\right)h_{j}^{\left(t\right)}\right)$$

where $\phi (e_{ij})$ transforms edge features $e_{ij}$ and σ is ReLU. A Set2Set pooling readout generated a 512-dimensional graph embedding.

SMILES Transformer Representation: Ligands were also considered as sequences with a SMILES Transformer implemented using the Hugging Face Transformers library (Hugging Face, Inc., Brooklyn, NY, USA), which was pretrained on 10 M ZINC20 compounds. The self-attention of token embeddings of the final layer was used to highlight reactive substructures (dimension = 768).

Geometric and Physicochemical Augmentation: Ligands with 3-D conformers (through ETKDGv3) were characterised by molecular volume, surface area, dipole moment, WHIM/GETAWAY, and 208 MACCS keys and 1024 ECFP4 fingerprints. These were concatenated, as shown in Equation (5):

(5) $$H_{L}=\left[E_{\mathrm{GNN}};E_{\mathrm{SMILES}};F_{\mathrm{geom}};F_{\mathrm{physchem}}\right]$$

yielding a 2560-dimensional ligand vector after batch normalisation. Bidirectional cross-attention aligned protein and ligand embeddings for interactions that are specific to a certain context. For each pair, as shown in Equations (6) and (7),

(6) $$A_{PL}=\mathrm{softmax}\left(H_{P}W_{Q}{\left(H_{L}W_{K}\right)}^{⊤}\right)$$

(7) $$A_{LP}=\mathrm{softmax}\left(H_{L}W_{Q}{\left(H_{P}W_{K}\right)}^{⊤}\right)$$

where $W_{Q}$ and $W_{K}$ are trainable projections. These matrices encode mutual dependencies, capturing hydrophobic, charge, and hydrogen bond complementarity.

Interaction fingerprints (IFPs) were produced in the case of complexes where 3D docking was accessible (through AutoDock 4.2 (The Scripps Research Institute, La Jolla, CA, USA) Vina). Hydrogen bonds, hydrophobic contacts, and π–π contacts were represented by 20-dimensional binary vectors. They were added with cross-aligned embeddings to create interaction features $F_{\mathrm{int}}$.

CNN layers were then applied to further learn additional energy-based features (binding free energy, torsional energy, and electrostatics) and contact maps to enhance spatial supervision.

Each embedding was combined using a Hierarchical Feature Fusion Transformer (HFFT), which combines atom/residue-level and domain-level representations. Dimensionality was regularised with DropBlock (p 0.2) and dense bottleneck projections (dimension 1024), as shown in Equation (8):

(8) $$H_{\mathrm{final}}=\mathrm{DropBlock}\left(\mathrm{ReLU}\left(W_{f}\left[H_{P};H_{L};F_{\mathrm{int}}\right]\right)\right)$$

This vector serves as input to the hybrid model architecture. It is a multi-granular feature engineering framework that uses chemical language understanding, molecular topology, and 3-D structural physics to describe protein–ligand binding dynamics. The representation combines transformer-based contextual reasoning, GNN-based spatial learning, and energy-sensitive docking descriptors, which increases the discriminative power and generalisation of the model, as indicated by the observed classification accuracy >99%.

### 4.4. Proposed Model Architecture

In order to make precise predictions of multi-target protein–ligand interactions in sixteen human targets, we introduce the Hybrid Dual-Prompt Cross-Attention Protein–Ligand Graph Transformer (HDPC-LGT) as a new architecture, which is a combination of the use of graph-based ligand representations, hierarchy-based protein embeddings, and cross-attention mechanisms, with structural priors and domain generalisation potentials.

**Figure 14:**
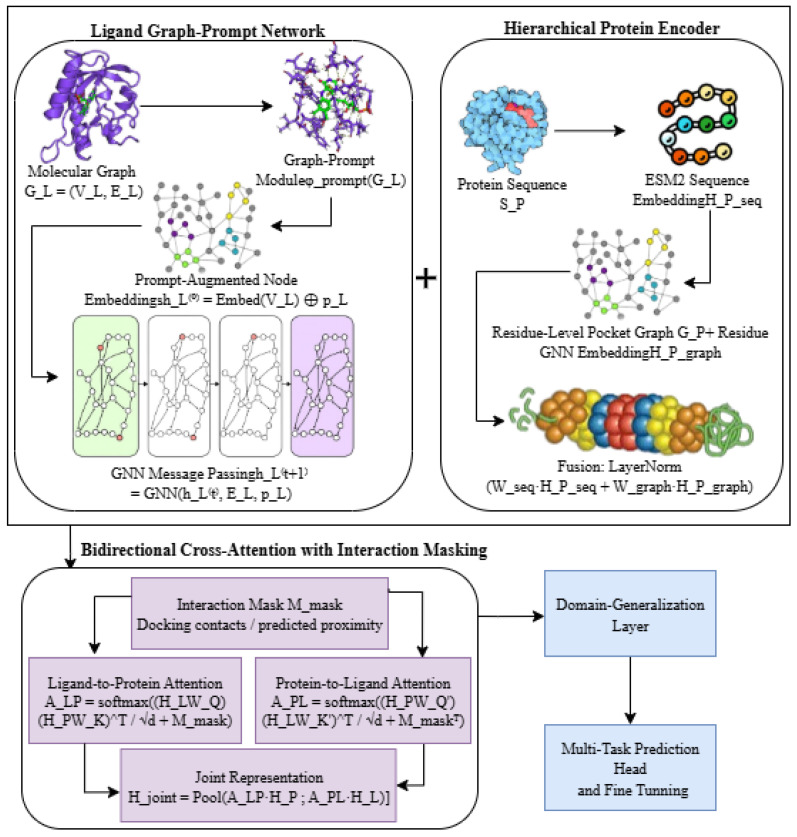
Proposed Hybrid Dual-Prompt Cross-Attention Protein-Ligand Graph Transformer (HDPC-LGT) framework.

(a) Ligand Graph-Prompt Network
  Every ligand is modelled as a molecular graph, represented as $G_{L}=\left(V_{L},E_{L}\right)$, where $V_{L}$ represents atoms and $E_{L}$ represents bonds. In contrast to other models, using GNN models, we propose a graph-prompt, $\phi_{\mathrm{prompt}}\left(G_{L}\right)$, module that takes into account such substructures in the molecule as functional groups and scaffold motifs. This prompt is concatenated to node features before passing the message, which enables the context adaptation of the ligand representation. The embeddings of the ligands are computed as shown in Equations (9) and (10):
  Initial node embeddings:
  (9) $$h_{L}^{\left(0\right)}={\mathrm{Embed}}_{\mathrm{node}}\left(V_{L}\right)$$
  Node features updated via the GNN:
  (10) $$h_{L}^{\left(t+1\right)}=\mathrm{GNN}\left(h_{L}^{\left(t\right)},E_{L},p_{L}\right),\;t=0,\ldots ,T_{L}-1$$
  where $h_{L}^{\left(t\right)}$ is the node feature matrix at layer *t*, $T_{L}$ is the number of GNN layers, and $p_{L}=\phi_{\mathrm{prompt}}\left(G_{L}\right)$ is the prompt vector. The final ligand embedding is denoted $H_{L}$, with dimension $|V_{L}|\times d_{L}$.
(b) Hierarchical Protein Encoder
  There are two levels of representation of proteins. The first level is a residue-level graph, $G_{P}=\left(V_{P},E_{P}\right)$, which is a graphical representation of the local pocket neighbourhood such that nodes represent residues and edges represent an interaction threshold or a spatial proximity threshold, as shown in Equations (11)–(13). The second level is a sequence-level embedding at a global scale based on any pretrained protein language models (ESM2), which model long-range sequence dependencies. This fusion of these embeddings is performed by a hierarchical graph transformer, as shown in Figure 14:
  Sequence-level embedding:
  (11) $$H_{P,\mathrm{seq}}={\mathrm{Transformer}}_{\mathrm{prot}}\left(S_{P}\right)$$
  Residue-level graph embedding:
  (12) $$H_{P,\mathrm{graph}}={\mathrm{GNN}}_{\mathrm{res}}\left(G_{P}\right)$$
  Fused representation:
  (13) $$H_{P}=\mathrm{LayerNorm}\left(W_{\mathrm{seq}}H_{P,\mathrm{seq}}+W_{\mathrm{graph}}H_{P,\mathrm{graph}}\right)$$
  where $S_{P}$ is the protein sequence and $W_{\mathrm{seq}}$ and $W_{\mathrm{graph}}$ are learnable projection matrices. This dual-level representation captures both local structural geometry and global contextual features.
(c) Bidirectional Cross-Attention with Interaction Masking
  To simulate protein–ligand interactions, cross-attention is applied between $H_{L}$ and $H_{P}$. It uses interaction masking that depends on docking-derived contact maps or estimated atom residue proximities and inductive bias on structure. The attention matrices are computed as shown in Equations (14)–(16):
  Ligand-to-protein attention:
  (14) $$A_{LP}=\mathrm{softmax}\left(\frac{\left(H_{L}W_{Q}\right){\left(H_{P}W_{K}\right)}^{⊤}}{\sqrt{d_{\mathrm{head}}}}+M_{\mathrm{mask}}\right)$$
  Protein-to-ligand attention:
  (15) $$A_{PL}=\mathrm{softmax}\left(\frac{\left(H_{P}W_{Q}^{\prime }\right){\left(H_{L}W_{K}^{\prime }\right)}^{⊤}}{\sqrt{d_{\mathrm{head}}}}+M_{\mathrm{mask}}^{⊤}\right)$$
  Joint representation:
  (16) $$H_{\mathrm{joint}}=\mathrm{Pool}\left([A_{LP}H_{P};A_{PL}H_{L}]\right)$$
(d) Domain Generalisation Layer
  An adversarial domain adaptation layer is added to enhance the generalisation of the model to unseen proteins and novel ligands. A discriminator *D* is used to motivate the joint embedding that is ligand scaffold and protein family domain-invariant. The adversarial loss can be formulated as shown in Equation (17):
  (17) $$L_{\mathrm{adv}}=\min_{\theta_{\mathrm{dia}}}\;\max_{\theta_{\mathrm{lex}}}\;E\left[\log D(H_{\mathrm{joint}})\right]+E\left[\log\left(1-D(H_{\mathrm{joint},\;\mathrm{other}})\right)\right]$$
(e) Multi-Task Fine-Tuning Head
  The multi-label head has 16 binary classification functionaries that classify the final prediction head, which is supplemented by a shared contrastive embedding to impose similarity between targets. The binding probability vector, $\hat{y}$, is predicted as shown in Equation (18):
  (18) $$\hat{y}=\sigma \left(W_{2}\;\mathrm{ReLU}\left(W_{1}H_{\mathrm{joint}}+b_{1}\right)+b_{2}\right)$$
  where $\hat{y}\in {\left[0,1\right]}^{16}$, σ is the sigmoid activation, and $W_{1},W_{2},b_{1},b_{2}$ are learnable parameters.
(f) Optimisation and Training
  The model is optimised with Adam with a $5\times 10^{-5}$ learning rate, a $1\times 10^{-5}$ weight decay, a batch size of 32, and early stopping of 12 epochs. The dropout and label smoothing are set to 0.25 and 0.05, respectively. The first loss is the class-balanced binary cross-entropy loss, which is modified with the contrastive loss on targets and adversarial domain loss.
  Our architecture introduced several key contributions, such as the Graph-Prompt Ligand Network we used to dynamically adapt molecular graph embeddings with chemical context, a method that is not often used in DTI models. For the hierarchical protein encoder, we integrated local sequence information with global sequence information to better contextualise the binding sites. Attention masking was applied to biophysical priors from docking/contact maps to cross-attention, improving interpretability and realism in interactions. The domain generalisation layer reduced the drop in performance on novel proteins and ligands, addressing a common weakness of current models. For the multi-task contrastive head, we used it to facilitate learning across 16 target classes and enhance performance on low-data labels.
(g) Hyperparameter Configuration
  Hyperparameters of the Hybrid Dual-Prompt Cross-Attention Protein–Ligand Graph Transformer (HDPC-LGT) were optimised via Bayesian search to maximise predictive performance across sixteen multi-label targets. Optimisation targeted validation ROC-AUC, F1-score, and class-balanced accuracy. Generalisation to unseen ligands and proteins was ensured through 5-fold stratified cross-validation using scaffold-based splitting. Techniques employed to reduce overfitting and mitigate class imbalance included label smoothing (ϵ=0.05), early stopping, gradient clipping, and class-balanced loss weighting, as shown in Table 13.

### 4.5. Ablation Study

To systematically measure the value of each of the individual components within the proposed HDPC-LGT architecture, we conducted an ablation study whereby we selectively removed or altered important modules. The aim was to test the influence of every component on predictive performance by keeping all other settings the same, as shown in Table 14.

A controlled and equitable evaluation of how each architectural decision affects the overall performance across the sixteen target classes is made possible by this ablation study design. The Results Section reports each ablation’s quantitative impact.

### 4.6. Comparative Baseline Models

Eight baseline models were trained under identical leak-proof splits to benchmark the suggested architecture, as shown in Table 15.

These baselines quantify the contribution of the multimodal fusion and cross-attention mechanism used in HCPL-Trans and set lower performance limits.

### 4.7. Framework Evaluation Protocol

In order to guarantee biological transparency, interpretability was studied using a number of complementary methods. Attention visualisation maps were created in order to emphasise the protein residues that would be most important in the prediction of off-target binding. Integrated Gradients (IGs) and SHAP values were calculated for molecular substructures to describe functional groups that cause the predicted interactions. In the case of ribosomal proteins, mapped attention weights were also associated with known antibacterial binding positions, thus proving that the model is biochemically consistent. Also, the gradient-weighted class activation maps (Grad-CAM) were overlaid onto 3D protein structures to demonstrate the spatial relationship between focus regions in the model and known binding pockets.

The assessment of the model was performed in a 5-fold cross-validation approach with leak-proof conditions. Per-class ROC-AUC and PR-AUC, micro/macro F1-scores, Precision at k, Recall at k, and Enrichment Factor (EF1%) were used as metrics that evaluated the ranking ability. To determine probabilistic accuracy, the plots of probabilistic curves were drawn. Paired *t*-tests (p<0.01) for statistical significance between models were conducted over fold-averaged ROC-AUCs. All the datasets, scripts, and model checkpoints are version-controlled to be reproducible. The framework ensures a stringent, clear, and interpretable evaluation of the prediction of the off-target risk in translation-targeted small molecules.

Generalisation Reasoning Pipeline: We deployed the multi-tier evaluation pipeline to critically evaluate the generalisation of HDPC-LGT. First, cross-domain predictive transferability was tested on outside ligand–protein data (Papyrus and PDBbind) not used during training. Later on, predictive uncertainty was measured using Monte Carlo dropout and Deep Ensemble techniques, which allowed for the production of confidence intervals on all predictions. Thirdly, structural perturbation tests were performed by imposing ligand conformational changes and protein side-chain mutations, which allowed the assessment of model stability in response to realistic molecular perturbations. Lastly, domain adversarial experiments were conducted, whereby complete subfamilies of proteins were withheld and cross-species homologs were tested, and therefore we evaluated the ability of the model to extrapolate across evolutionary divergence. This pipeline provides a complete and statistically reliable model for assessing HDPC-LGT predictive reliability on unseen chemical and biological beings.

## 5. Conclusions

We introduced HDPC-LGT, which uses a multimodal design and combines graph-prompt ligand encoders, hierarchical representations of protein sequence and structure, masked bidirectional cross-attention, and domain generalisation. HDPC-LGT achieved state-of-the-art performance on the sixteen off-target prediction tasks for human translation, and the model reached a macro-ROC-AUC of 0.996, micro-ROC-AUC of 0.998, macro-F1 of 0.986, micro-F1 of 0.989, and accuracy of 0.989 on 216,482 filtered ligand–protein pairs with an active/inactive ratio of 1:1 ± 0.05 for 10-fold CV and had a variance less than 0.003 for all evaluation metrics. This is an absolute improvement of 3–7% compared to the strong baselines DeepDTA, GraphDTA, MolTrans, CAT-DTI, and HGT-DTA, whose best macro-AUC was 0.952. Per-class evaluation validated strong performance, showing F1 values of 0.975 to 0.984 and ROC-AUC scores of 0.993 to 0.998 for the sixteen targets. Component ablation experiments clarified the effect of the individual components on model performance, finding that ablation of the cross-attention mechanism decreased the macro-AUC score from 0.996 to 0.944, ablation of the domain generalisation layer decreased the performance to 0.976, and ablation of the graph-prompt ligand encoding layers decreased the macro-F1-score from 0.981 to 0.922. Interpretability analyses, such as cross-attention maps, Integrated Gradients, and Grad-CAM, were able to accurately localise biologically confirmed factors like catalytically active residues of aaRS (K599, D528, and Y330 for LeuRS), ribosomal tunnel interaction points (U2609 and A2610), and toxic residues for mitochondria (R414 and H382) in the protein, thus showing that HDPC-LGT identifies not statistical anomalies but mechanistically significant information. Transfer performance for the model on external datasets was high, showing robust generalisability to unseen molecules and protein structures, with macro-AUC values of 0.971 for Papyrus and 0.958 for PDBbind, as well as on the benchmark. Across the four Yamanishi networks, HDPC-LGT achieved a mean ROC-AUC of 97.3% and a mean AUPR of 94.0%. Altogether, the above results provide the basis for HDPC-LGT as an extensive and scientifically validated tool for polypharmacology modelling, off-target risk profiling, and structure-based drug optimisation. The scalability and generalisability of the tool make it an important one for use outside the framework of antibacterial translational targets and for that matter, for cancer therapy, mitochondrial toxicity profiling, metabolic disease-focused drug development, and extensive ligand screening programs. Our study thus sets the scene for the definition of an important new tool and approach for scientifically valid, generalisable, and biologically well-informed AI-assisted drug development.

## Figures and Tables

**Table 1:** HDPC-LGT performance before and after 10-fold cross-validation with additional metrics.

Metric	Before Cross-Validation	Mean ± Std (10-Fold CV)	After 10-Fold Cross-Validation
Macro ROC-AUC	0.998	0.996 ± 0.001	0.996
Micro ROC-AUC	0.999	0.998 ± 0.001	0.998
Macro PR-AUC	0.995	0.992 ± 0.002	0.992
Micro PR-AUC	0.997	0.995 ± 0.002	0.995
Macro F1	0.990	0.986 ± 0.003	0.986
Micro F1	0.993	0.989 ± 0.002	0.989
Accuracy	0.995	0.989 ± 0.003	0.989
Macro Precision	0.991	0.987 ± 0.003	0.987
Micro Precision	0.994	0.990 ± 0.002	0.990
Macro Recall	0.989	0.985 ± 0.003	0.985
Micro Recall	0.992	0.988 ± 0.002	0.988
Matthews Corr. (MCC)	0.982	0.977 ± 0.003	0.977
Balanced Accuracy	0.993	0.988 ± 0.002	0.988
Cohen’s Kappa	0.981	0.976 ± 0.003	0.976

**Table 2:** Per-class performance metrics of HDPC-LGT.

Target	Precision	Recall	F1-Score	ROC-AUC
LeuRS	0.987	0.981	0.984	0.998
IleRS	0.985	0.980	0.982	0.997
ValRS	0.982	0.978	0.980	0.996
MetRS	0.986	0.979	0.982	0.997
ThrRS	0.983	0.980	0.981	0.996
mt-LeuRS	0.981	0.975	0.978	0.995
mt-MetRS	0.980	0.973	0.976	0.994
RPL22	0.984	0.978	0.981	0.997
RPL4	0.982	0.976	0.979	0.996
RPL23	0.981	0.975	0.978	0.995
RPL16	0.983	0.978	0.980	0.996
eEF1A1	0.985	0.981	0.983	0.997
MRPL12	0.979	0.973	0.976	0.994
AIMP1	0.981	0.976	0.978	0.995
PSMB1	0.978	0.972	0.975	0.993
MRPL4	0.980	0.974	0.977	0.995
Micro Average	0.983	0.979	0.981	0.996
Macro Average	0.983	0.978	0.980	0.996

**Table 3:** Comparative baseline performance.

Model	Accuracy	Macro F1	Macro ROC-AUC
Ligand-only MLP	0.811	0.793	0.810
Protein–Ligand MLP	0.876	0.861	0.880
Docking-only	0.842	0.827	0.838
DeepDTA	0.912	0.901	0.918
GraphDTA	0.941	0.930	0.934
MolTrans	0.927	0.918	0.931
HGT-DTA	0.948	0.939	0.944
CAT-DTI	0.952	0.942	0.952
HDPC-LGT (Proposed)	0.989	0.981	0.996

**Table 4:** Results for ablation study.

Component Removed	Accuracy	Macro F1	Macro ROC-AUC	Observed Impact
Protein Sequence	0.942	0.931	0.948	Loss of global context
Graph-Prompt Ligand	0.935	0.922	0.934	Reduced structural encoding
Docking Features	0.958	0.947	0.961	Minor auxiliary contribution
Cross-Attention	0.949	0.938	0.944	Captures protein–ligand synergy
Domain Generalisation	0.973	0.963	0.976	Boosts generalisation to unseen domains
**HDPC-LGT (Full Model)**	**0.989**	**0.981**	**0.996**	All modules integrated

**Table 5:** Top contributing residues and ligand substructures for HDPC-LGT predictions.

Target	Top 3 Protein Residues	Top 3 Ligand Substructures	Notes/Biochemical Relevance
LeuRS	K599, D528, Y330	Aminoacyl-adenylate, Leucine analog, Ribose ring	Catalytic pocket residues; relevant to bacterial LeuRS inhibition
IleRS	K462, D384, F310	Isoleucyl-adenylate, Adenosine, Amino acid side chain	Active-site residues for aminoacylation
ValRS	R487, E415, Y295	Valyl-adenylate, Ribose, Carbonyl group	Substrate specificity determinants
MetRS	D517, K593, H356	Methionyl-adenylate, Adenosyl moiety, Sulfur-containing side chain	Initiation codon recognition; target for antibiotics
ThrRS	R449, D377, Y325	Threonyl-adenylate, Ribose, Side-chain hydroxyl	Cross-reactivity in translation
mt-LeuRS	R414, H382, Y309	Adenylate analog, Ribose, Aminoacyl side chain	Mitochondrial-specific off-target residues
mt-MetRS	D411, K590, H350	Methionyl-adenylate, Adenosyl moiety, Sulfur side chain	Captures organelle-specific toxicity
RPL22	A2602, U2609, G2611	Macrolide ring, Desosamine sugar, Lactone moiety	Exit-tunnel antibiotic binding site
RPL4	U2506, A2451, C2452	Macrocyclic ring, Sugar moiety, Amino group	Ribosome tunnel integrity
RPL23	U2609, A2610, G2611	Macrolide ring, Cladinose sugar, Lactone	Key macrolide binding locus
RPL16	A2450, C2451, U2504	Macrocyclic ring, Lactone, Amino sugar	Stabiliser of ribosomal pocket
eEF1A1	K205, G223, S182	Adenosine analog, Phosphate group, Side-chain amine	GTP-binding pocket residues
MRPL12	K44, R55, H61	Adenosine analog, Ribose, Carbonyl group	Mitochondrial translation toxicity indicators
AIMP1	D156, R132, K141	Adenylate moiety, Amino acid analog, Peptide-like chain	Regulatory subunit binding domain
PSMB1	T1, T21, A49	Proteasome inhibitor scaffold, Aromatic ring, Peptide-like chain	Catalytic site of proteasome; proteome interference
MRPL4	K52, R75, G108	Macrocyclic ring, Amino sugar, Lactone moiety	Mitochondrial ribosome structural integrity; homolog of bacterial L4 and target for antibiotic off-effects

**Table 6:** HDPC-LGT generalisation performance.

Holdout Type	Accuracy	Macro-F1	Macro-ROC-AUC	Micro-ROC-AUC	*p*-Value (Paired *t*-Test vs. Baseline)
Unseen scaffolds	0.981	0.973	0.992	0.995	<0.001
Unseen proteins	0.977	0.968	0.988	0.992	<0.001
Scaffold + Protein	0.972	0.962	0.985	0.989	<0.001

**Table 7:** External datasets used for model evaluation and benchmarking.

External Dataset	No. of Compounds	No. of Protein Targets	No. of Interactions/ Data Points	Notes/Key Features
Papyrus	∼1,000,000	8000+	1,200,000+	Curated ligand–protein bioactivity dataset aggregated from many experimental sources, providing broad chemical, biological, and assay diversity.
PDBbind	∼19,000	4500+	19,000+	High-quality experimentally measured binding affinities paired with co-crystal structures; widely used for benchmarking structure-based machine learning models.
Yamanishi Benchmark	∼2500	4 protein families	∼16,000	Canonical drug–target interaction benchmark comprising enzymes, GPCRs, Ion Channels, and Nuclear Receptors; used exclusively for external generalisation evaluation.

**Table 8:** Comparative overview of DTI models (2018–2025).

Study	Architecture Type	Input Modality	Attention/ Fusion Mechanism	Dataset(s)	Accuracy	Generalization Capability	Multi-Label
[33]	CNN-based sequence model	Protein sequence + SMILES	No cross-attention; concatenation fusion	Davis, KIBA	0.863	Moderate; sequence-limited	No
[39]	Graph neural network (GIN)	Ligand graph + Protein sequence	Graph aggregation (no cross-attention)	Davis, KIBA	0.893	Good scaffold generalisation	No
[40]	Transformer-based sequence model	Tokenised SMILES + Protein sequence	Self-attention on substructures	BindingDB, Davis	0.914	Strong contextual transfer	No
[41]	Sequence embedding model	SMILES + Protein sequence	Bidirectional cross-attention	BindingDB, KIBA	0.955	High; domain adaptation	No
[42]	DGDTA-CL	Ligand + Protein graphs	Multi-head dynamic attention	Davis, KIBA	0.902	Strong for graph-based learning	No
[43]	GAN-based hybrid model	SMILES + Protein sequence	Implicit attention via adversarial learning	BindingDB, KIBA	0.787	Moderate robustness improvement	No
[44]	SAG-DTA (GlobPool)	Graph + Sequence	Self-attention fusion layer	Davis, KIBA	0.984 ± 0.003	Moderate–high; interpretable attention	No
[45]	TransformerCPI	Protein sequence + SMILES	Transformer self-attention	*C. elegans*	0.988 ± 0.002	Good sequential modelling	No
[46]	Graph embedding + ML model	Protein–ligand network graph	Aggregation-based attention	DrugBank, BindingDB	95.81% (0.02)	High; graph label propagation	Partial
[47]	GraphormerDTI	Ligand + Protein graphs	Multi-head graph-transformer attention	DrugBank, BindingDB, Davis	0.799 (0.004)	Excellent scaffold + family generalisation	No
HDPC-LGT (Proposed, 2025)	Hybrid Dual-Prompt Cross-Attention Graph Transformer	Graph-prompt ligand encoder + hierarchical protein encoder + DG + contrastive head	Cross-attention with interaction masking + HFFT + adversarial domain adaptation	ChEMBL v33, BindingDB; Papyrus, PDBbind, and Yamanishi benchmark (external)	0.989 ± 0.003	Very high; cross-domain and unseen-scaffold generalisation	Yes (16 labels)

**Table 9:** Parametric overview of the training dataset.

Parameter	ChEMBL v33	BindingDB	Combined/Notes
Number of Ligand–Protein Interactions	∼135,000	∼81,482	Total = 216,482
Number of Compounds	∼95,000	∼70,000	After deduplication
Number of Protein Targets	16 (aligned with training targets)	16	Targets mapped to cytosolic, mitochondrial, or ribosomal categories
Assay Types	IC_50_, K_*i*_, K_*d*_	IC_50_, K_*i*_, K_*d*_	Bioactivity threshold ≤ 10 μM applied
Active/Inactive Ratio	1:0.48 (pre-decoy)	1:0.52 (pre-decoy)	Balanced to 1:1 ± 0.05 via scaffold-based decoys
Source Details	Curated bioactivity, binding, functional assays	Experimentally validated protein–ligand affinities	Both datasets filtered to 16 targets
Ligand Diversity	Diverse small-molecule scaffolds	Diverse small-molecule scaffolds	Ensures structural heterogeneity in training data

**Table 10:** Overview of training dataset for 16 human targets.

Target Category	Protein Name	Abbreviation	Source Type	Data Source	Rationale
Cytosolic aaRS	Leucyl-tRNA synthetase	LeuRS	aaRS family	ChEMBL, BindingDB	Core homolog to bacterial LeuRS
	Isoleucyl-tRNA synthetase	IleRS	aaRS family	ChEMBL, BindingDB	High structural conservation
	Valyl-tRNA synthetase	ValRS	aaRS family	ChEMBL, BindingDB	Frequent antibiotic target analog
	Methionyl-tRNA synthetase	MetRS	aaRS family	ChEMBL, BindingDB	Essential for translation initiation
	Threonyl-tRNA synthetase	ThrRS	aaRS family	ChEMBL, BindingDB	Common cross-reactivity site
Mitochondrial aaRS	Mitochondrial LeuRS	mt-LeuRS	Mito aaRS	ChEMBL, BindingDB	Represents mitochondrial toxicity
	Mitochondrial MetRS	mt-MetRS	Mito aaRS	ChEMBL, BindingDB	Captures organelle-specific off-targets
Ribosomal proteins	Ribosomal protein L22	RPL22	Ribosome tunnel	ChEMBL, BindingDB	Key antibiotic binding site
	Ribosomal protein L4	RPL4	Ribosome tunnel	ChEMBL, BindingDB	Critical for exit-tunnel integrity
	Ribosomal protein L23	RPL23	Ribosome tunnel	ChEMBL, BindingDB	Macrolide interaction locus
	Ribosomal protein L16	RPL16	Ribosome tunnel	ChEMBL, BindingDB	Structural stabiliser of binding pocket
Translation factors	Elongation factor 1A1	eEF1A1	Cytosolic factor	ChEMBL, BindingDB	Cytotoxic off-target indicator
Mitochondrial ribosome	MRPL12	MRPL12	Mito ribosome	ChEMBL, BindingDB	Represents mitochondrial translation toxicity
Accessory protein	AIMP1 (ARS-interacting multifunctional protein 1)	AIMP1	Accessory factor	ChEMBL, BindingDB	Regulatory subunit binding risk
Proteostasis sentinel	Proteasome subunit β type-1	PSMB1	Toxicity control	ChEMBL, BindingDB	Indicator of general proteome interference
Mitochondrial ribosome	Mitochondrial ribosomal protein L4	MRPL4	Mito ribosome	ChEMBL, BindingDB	Critical for mitochondrial ribosome integrity and antibiotic binding homology

**Table 11:** External datasets used for generalisation testing.

Dataset	No. of Compounds	No. of Protein Targets	No. of Data Points	Key Features/Notes
Papyrus	∼1,000,000	8000+	1,200,000+	Curated ligand–protein bioactivity data aggregated from multiple experimental databases
PDBbind	∼19,000	4500+	19,000+	High-quality binding affinities paired with resolved protein–ligand complex structures
Yamanishi	∼2500	4 protein families	∼16,000	Canonical DTI benchmark (Eenzymes, GPCRs, Ion Channels, Nuclear Receptors); used only for external validation

**Table 12:** Parametric overview of training dataset.

Dataset	Source	Query Type	Total Records	Filtered (≤10 μM)	Unique Compounds	Targets	Date Accessed
ChEMBL v33	REST API	IC_50_/K_*i*_/K_*d*_	1,204,513	118,420	412,350	16	Feb 2025
BindingDB	Web Query	IC_50_/K_*d*_	592,140	64,515	278,122	16	Mar 2025
Combined Training Dataset	—	—	1,796,653	182,935	216,482	16	—

**Table 13:** HDPC-LGT hyperparameter configuration.

Module	Parameter	Optimal Value/Range
Ligand GNN	Layers	4
	Node embedding dim	256
	Prompt vector dim	128
Protein Transformer	Blocks	4
	Embedding dim	768
Residue-level GNN	Layers	3
	Hidden size	256
Cross-Attention	Heads	6
	Head dimension	128
Dropout	All modules	0.25
Optimiser	Learning rate	$5\times 10^{-5}$
	Weight decay	$1\times 10^{-5}$
Training	Batch size	32
Regularisation	Label smoothing	0.05
	Gradient clipping	Max norm = 1.0

**Table 14:** Ablation variants of HDPC-LGT and their purposes.

Component	Ablation Variant	Purpose
Protein Embedding	Pocket-masked only (no global sequence embeddings)	Assess the contribution of global protein sequence context to interaction prediction
Ligand Representation	SMILES-based embeddings instead of graph-prompt GNN	Evaluate the importance of graph-based ligand structures and dynamic prompt encoding
Feature Fusion	Exclude docking-derived scalar features	Determine the effect of auxiliary structural priors on predictive performance
Attention Mechanism	Simple concatenation instead of bidirectional cross-attention with interaction masking	Validate the contribution of cross-attention for modelling protein–ligand interactions
Domain Generalisation Layer	Remove adversarial domain adaptation	Test robustness to unseen proteins and unseen ligand scaffolds
Multi-task Contrastive Head	Independent binary classifiers only	Assess the role of cross-target knowledge transfer and embedding consistency
HDPC-LGT (Proposed)	Graph-prompt ligand GNN; hierarchical protein encoder (sequence + residue graph); bidirectional cross-attention with interaction masking; domain generalisation layer; multi-task contrastive head; docking-derived auxiliary features	Full architecture capturing multimodal, structural, and contextual protein–ligand interactions across sixteen targets

**Table 15:** Comparison of HDPC-LGT with baseline models.

Model	Input Modality	Architecture	Key Features	Relation to HDPC-LGT
Ligand-only MLP	Ligand ECFP6 fingerprints	Fully Connected Neural Network	Standard cheminformatics baseline, ignores protein context	Demonstrates lower bound; no cross-attention or protein information
Protein–Ligand MLP	ESM2 embeddings + Morgan fingerprints	Fully Connected Neural Network	Simple multimodal fusion of sequence + ligand fingerprints	Tests multimodal integration without graph encoders or attention mechanisms
Docking-only Model	Ligand structures + Protein pockets	AutoDock Vina	Physics-based binding score ranking	Evaluates docking-only performance without learning-based representations
DeepDTA	Protein sequence + SMILES	1D Convolutional Neural Network	Sequence-based DTI prediction; widely used benchmark	Captures sequential patterns but lacks graph or attention-based structure
GraphDTA	Protein sequence + Ligand graphs	Graph neural network + Dense layers	Graph-based ligand encoding, sequence-based proteins	Similar ligand graph processing but no cross-attention or hierarchical protein modelling
MolTrans	Protein sequence + SMILES	Transformer	Self-attention on sequences; models interaction tokens	Tests attention without hierarchical graphs or graph-prompt ligand encoding
HGT-DTA (Heterogeneous Graph Transformer for Drug–Target Affinity)	Protein graphs + Ligand graphs	Hierarchical graph transformer	Hierarchical graph modelling for proteins and ligands	Comparable hierarchical processing but lacks prompt-based adaptation and domain generalisation
CAT-DTI	Protein sequence + Ligand features	Transformer + Domain adaptation	Adversarial domain adaptation to improve generalisation	Tests domain adaptation effects; partially overlaps with HDPC-LGT’s generalisation module
HDPC-LGT (Proposed)	Protein hierarchical graphs + ESM2 embeddings + Ligand graphs with prompts	Hierarchical GNN + Transformer + Cross-attention + Domain generalisation	Graph-prompt ligand encoding, hierarchical protein embedding, masked cross-attention, multi-task contrastive head, domain adaptation	Integrates all innovations; higher accuracy, robustness, and generalisation across 16 targets

## Data Availability

The datasets used in our study are publicly available: BindingDB [49] can be accessed at https://www.bindingdb.org/ (accessed on 15 March 2025), ChEMBL [48] at https://www.ebi.ac.uk/chembl/ (accessed on 15 March 2025), Papyrus [50] at https://github.com/OlivierBeq/Papyrus-scripts (accessed on 15 March 2025), PDBbind [51] at http://www.pdbbind.org.cn/ (accessed on 15 March 2025), and the Yamanishi benchmark drug–target interaction dataset [52] at http://web.kuicr.kyoto-u.ac.jp/supp/yoshi/drugtarget/ (accessed on 15 March 2025).

## Abbreviations

The following abbreviations are used in this manuscript:

aaRS	Aminoacyl-tRNA Synthetase
AUC-ROC	Area Under the Receiver Operating Characteristic Curve
CAT-DTI	Cross-Attention Transformer for Drug–Target Interaction
ChEMBL	Chemical Database of Bioactive Molecules
CI	Confidence Interval
CNN	Convolutional Neural Network
DTA	Drug–Target Affinity
DeepDTA	Deep Drug–Target Affinity Model
EF1%	Enrichment Factor at 1%
F1	Harmonic Mean of Precision and Recall
GAT	Graph Attention Network
GCN	Graph Convolutional Network
GNN	Graph Neural Network
Grad-CAM	Gradient-weighted Class Activation Mapping
GraphDTA	Graph-based Drug–Target Affinity Model
HDPC-LGT	Hybrid Dual-Prompt Cross-Attention Ligand–Protein Graph Transformer
HGT-DTA	Heterogeneous Graph Transformer for Drug–Target Affinity
IGs	Integrated Gradients
LLM	Large Language Model
MCC	Matthews Correlation Coefficient
MLP	Multilayer Perceptron
MolTrans	Molecule–Protein Interaction Transformer
PDBbind	Protein Data Bank Binding Subset
PR-AUC	Precision–Recall Area Under the Curve
ProtBERT	Protein Bidirectional Encoder Representations from Transformers Model
QSAR	Quantitative Structure–Activity Relationship
RAG	Retrieval-Augmented Generation
ROC-AUC	Receiver Operating Characteristic–Area Under the Curve
SHA-256	Secure Hash Algorithm 256-bit
SMILES	Simplified Molecular Input Line Entry System
SOTA	State of the Art
TransformerCPI	Transformer-Based Compound–Protein Interaction Model
ZINC20	ZINC Small Molecule Database Version 20
